# ECMO: Improving our Results by Chasing the Rabbits

**DOI:** 10.5935/1678-9741.20150088

**Published:** 2015

**Authors:** Luiz Fernando Canêo, Rodolfo A. Neirotti

**Affiliations:** 1 Chairman of Extracorporeal Life Support Organization - Latin America Chapter (ELSO - LATAM) and Brazilian Society of Cardiovascular Surgery, São Paulo, SP, Brazil; 2 Clinical Professor of Surgery and Pediatrics, Emeritus Michigan State. Honorary member of Brazilian Society of Cardiovascular Surgery, Brookline, MA, USA

**Keywords:** Extracorporeal Membrane Oxygenation, Heart Defects, Congenital, Cardiovascular Surgical Procedures, Technology

## Abstract

As Marcelo Giugale published in the Financial Times, Latin America, on the whole, has not excelled at innovation - doing the same things in a new and better way or at doing new things. It has been slow to acquire, adopt and adapt technologies by this time available in other places^[1]^. Although extracorporeal membrane oxygenation (ECMO) is not a new technology, its use in Latin America is not widespread as needed. Furthermore, we still have a number centers doing ECMO, not reporting their cases, lacking a structured training program and not registered with the extracorporeal life support organization (ELSO). With this scenario, and accepting that ECMO is the first step in any circulatory support program, it is difficult to anticipate the incorporation of new and more complex devices as the technologically advanced world is currently doing. However, the good news is that with the support of experts from USA, Europe and Canada the results in Latin America ELSO'S centers are improving by following its guidelines for training, and using a standard educational process. There is no doubt that we can learn a great deal from the high velocity organizations - the rabbits - whom everyone chases but never catches, that manage to stay ahead because of their endurance, responsiveness, and their velocity in self-correction^[2]^.

**Table:** 

**Abbreviations, acronyms & symbols**	
ECMO	= Extracorporeal membrane oxygenation
ELSO	= Extracorporeal life support organization
PMP	= Poly-methylpentene

*"Insanity is doing something over and over again and expecting a
different result."*
Albert Einstein

## CONCEPTS AND PRACTICES

Since its beginning 37 years ago, the use of extracorporeal membrane oxygenation as an advanced life support therapy has evolved from the complex circuit with a roller pump and a silicon membrane to the simplified circuit with a centrifugal pump and a polymethylpentene membrane in use nowadays.

Although, Extracorporeal Membrane Oxygenation (ECMO) could be regarded as an "old dog" in the developed countries, its current form is still considered a new technology in Latin America. The Extracorporeal Life Support Organization (ELSO) was founded in 1978, as an international consortium with the mission of maintaining a registry of the use of extracorporeal membrane oxygenation in the registered centers. Nevertheless, looking at the map of the centers registered in ELSO, in Latin America there were centers only in Chile and Colombia. In fact, the Pontificia Universidad Católica de Chile, located in Santiago, was the first center to join ELSO, in 2003. Then, we can say that 37 years after the first successful case reported in USA, ECMO was not a usual procedure in most of the Latin American countries. A survey, made 3 years ago and presented during the 25^th^ ELSO meeting in Ann Harbor, Michigan, USA^[3]^, showed that there is a gap between the starting of using ECMO, the registration of the center in ELSO, and sending their data to the international registry. ELSOs' international consortium is nucleating an important number of health care professionals and scientists dedicated to develop and to evaluate ECMO as a support of the failing cardiopulmonary system. In addition, the organization provides educational programs for active centers as well as for the broader medical and lay communities. Therefore, it is fair to say that those that are not part of this society are most probably not following its guidelines and principles, and possibly not doing ECMO in the correct way.

Historically, in Latin America, ECMO was strongly related to cardiac surgery. In countries like Brazil, it was often considered an extension of the regular cardiopulmonary bypass that is transferring the patient to the intensive care unit connected to the pump with the usual hollow fiber oxygenator. Due this misconception, the results with ECMO were not good for many of us, and for some, it is still a way to postpone death.

After the H1N1 outbreak, others specialties, especially clinical intensivists working in non-cardiac centers, showed a great interest in learning how to use this therapy in their daily practice. Moreover, the new demand for V-V ECMO for respiratory support stimulated the interest in training people not previously familiar with this technology. Following ELSO's guidelines for training, centers from Chile, Colombia and Brazil started a standard education process, with the support of experts from USA, Europe and Canada. Furthermore, the foundation of the Latin America chapter, in 2012, facilitated the partnership with specialists of the developed world - the rabbits - as well as to bring trainers to our recently joined ELSO centers, to reproduce the same courses they used to have in their countries.

The first ECMO Specialist Training Course^[4]^ was held 3 years ago in Brazil at the Heart Institute, University of Sao Paulo Medical School, with a similar format of those courses offered in every center in the USA and Canada. It combined lectures with hands on, as well as high fidelity simulation teaching focused on the ECMO circuit and its clinical applications.

Although, our experience initiated in the late 90's, only very few members of the multidisciplinary team - particularly the direct patient caregivers - demonstrated familiarity with the materials and resources. This scenario has completely changed with the continued training of these professionals and the subsequent improvement of the results showed in a recently published paper of our center^[5]^. Our outcomes improved by using a 1:1 ratio model, where patient and ECMO are both handled by a single bedside nurse without a dedicated core team. Before training, the probability of weaning and survival after post-cardiotomy ECMO was 25% with only 5.5% discharge rate. After training, the therapy was used in 20 patients, and weaning was possible in 17 (85%), with 9 (45%) hospital discharges (*P* <0.01). Similar improvements, mainly related to training, occurred in 4 centers in Chile, 6 in Argentina, 2 in Colombia, 2 in Mexico, 3 in Peru and 1 in Costa Rica. These centers joined ELSO, they are following the organization's guidelines and they are reporting their data to the organization's database. In our continent, we find 3 times more papers in the last four years when we compare the number of publications by Latin American authors with the time we had no registered centers in ELSO. Altogether, training is improving the quality and safety demonstrating the importance of doing ECMO in the right way. Clinica las Condes, in Santiago, Chile, received the award of Center of Excellence^[6]^ for their high quality standard, and became the first one recognized by ELSO in Latin America. They have done more than 53 transports in patients on ECMO - among of the largest volumes in the world. Centers of development, with the necessary funds and manpower for research to generate and accumulate know-how can advance a diversity of new procedures requiring their use for a more efficient treatment, cost benefit ratio, and sustainability of care. These centers, can then disseminate the new knowledge, minimizing or even eliminating the learning curve as well as producing policies for the future of the specialty^[7]^.

Even though, we are seeing a reorganization of the people around us and some are following these principles, trying to achieve excellence in the multiple centers and contexts around the world will require time and hard work. As an example, nowadays there are nine ELSO centers in Brazil that are supposed to report their data and results. However, the ELSO's data and the numbers of poly-methylpentene (PMP) membrane sold in the country show a huge decentralization and a great number of unreported procedures, probably conducted by non-trained personnel. It is estimated that the number of ECMO runs reported to ELSO represents only 10% of the PMP membranes sold, clearly reflecting that we have main centers not registered in the organization, not reporting their data, not following the guidelines, and without an established training program. In this scenario, is understandable and even acceptable to assume that many consider this therapy "experimental", without proven results, not cost-effective and closely related to death.

ECMO requires a high-level of technical and non-technical skills, associated with an interdisciplinary teamwork, with the difficulties to accomplish the latter in the Latin American context. Looking to the future and talking about an advanced circulatory support program, ECMO is the first step, the kindergarten. Since the beginning, when we debate about ECMO, or even other complex technologies, the blame always goes to our economic situation - they are too expensive!

## WHERE ARE WE GOING?

Unfortunately, in our health system, it is often preferable to reach a large number of people with sub-optimal care than to reach fewer people with a more sophisticated, and therefore more expensive care. Cardiac and respiratory support will evolve with the incorporation the new devices, and will need centers of excellence with a well-trained people and the best possible basic education working as a team. Because the adoption of new technology should be followed by good results and cost-effectiveness, the health priorities in developing countries, and the status of their health systems are likely to be the limiting factors in accomplishing a widely available care. International partnership is important to train people and to achieve better results with new tools. "Nonetheless, foreign assistance should be adjusted to the local context avoiding dropping a replica of a proven model into an obsolete system - the name for the practice behind the problem is isomorphic mimicry. Unless resident agents work to give it a life of its own, it remains a replica"^[8]^.

What concerns us most is that there is still a lot more to come, demanding major changes in the society, the economy and the health care. The best way to predict the future is to invent it - this is our real challenge!

**Table:** 

**Authors' roles & responsibilities**	
LFC	Final manuscript approval; manuscript writing or critical review of its content
RAN	Final manuscript approval; manuscript writing or critical review of its content
